# Multi-Province Outbreak of Acute Gastroenteritis Linked to Potential Novel Lineage of GII.17 Norovirus in Argentina in 2024

**DOI:** 10.3390/v17020223

**Published:** 2025-02-05

**Authors:** Karina A. Gomes, Juan I. Degiuseppe, Paula M. Morón, Karina A. Rivero, Christian Barrios Matthieur, Carolina Paladino, Facundo G. Cuba, María S. Haim, Tomás J. Poklépovich Caride, Juan A. Stupka

**Affiliations:** 1 Laboratory of Viral Gastroenteritis-ANLIS “Dr. Carlos G. Malbrán”, Ciudad Autónoma de Buenos Aires 1281, Argentina; jdegiuseppe@anlis.gob.ar (J.I.D.); pmoron@anlis.gob.ar (P.M.M.); krivero@anlis.gob.ar (K.A.R.); cbarrios@anlis.gob.ar (C.B.M.); jstupka@anlis.gob.ar (J.A.S.); 2 National Center of Genomics and Bioinformatics-ANLIS “Dr. Carlos G. Malbrán”, Ciudad Autónoma de Buenos Aires 1281, Argentina; cpaladino@anlis.gob.ar (C.P.); fcuba@anlis.gob.ar (F.G.C.); mhaim@anlis.gob.ar (M.S.H.); tjcaride@anlis.gob.ar (T.J.P.C.)

**Keywords:** norovirus GII.17, gastroenteritis, outbreak

## Abstract

Noroviruses represent one of the leading causes of outbreaks and sporadic acute gastroenteritis (AGE) cases across all age groups. Although the GII.4 norovirus has been identified as the primary cause of most AGE outbreaks, the transient predominance of other genotypes has been reported globally. In this study, we describe a multi-province AGE outbreak caused by a potential new lineage of norovirus GII.17[P17], which has been recently detected at a high incidence in the United States and Europe. An amino acid analysis of the major viral capsid protein revealed several substitutions in the hypervariable region compared to strains circulating in the mid-2010s, which could play a key role in immune evasion. This is the first report of the detection of these viruses in the Southern Hemisphere, underscoring the importance of maintaining active genomic surveillance in the context of increasing numbers of acute gastroenteritis outbreaks.

## 1. Introduction

Norovirus is recognized as the leading etiological agent responsible for gastroenteritis outbreaks across all age groups globally [1]. Following the successful implementation of rotavirus vaccines, norovirus has emerged as the primary cause of gastroenteritis in children under five years old. Moreover, norovirus has a substantial impact on elderly and immunocompromised populations [2]. Current estimates suggest that norovirus infections cause approximately 200,000 deaths worldwide annually [3]. Norovirus’s high transmissibility is attributed to its extremely low infectious dose, environmental persistence, and significant viral shedding from infected individuals.

Norovirus has a single-stranded RNA genome approximately 7.5 kb in length, divided into three open reading frames (ORF1, ORF2, and ORF3). ORF1 encodes a polyprotein that is cleaved into six non-structural proteins, one of which is RNA-dependent RNA polymerase (RdRp). ORF2 encodes the major capsid protein, VP1, while ORF3 encodes the minor capsid protein, VP2. Noroviruses are classified into 10 genogroups, with genogroups I (GI), II (GII), IV (GIV), VIII (GVIII), and IX (GIX) known to infect humans. Each genogroup is further subdivided into distinct genotypes and variants based on VP1 sequence diversity. Several studies have identified the ORF1/ORF2 overlapping region as a site of frequent recombination. Also, the 3’ end of ORF1 encodes for RdRp, which, based on sequence diversity, can be classified into multiple types, leading to dual genotyping [4,5,6]. The GII.4 genotype is responsible for most norovirus outbreaks of gastroenteritis worldwide and is a significant cause of sporadic gastroenteritis cases in children under five years of age [7,8]. Norovirus from genotype GII.17 has been described to circulate since the 1970s; however, such circulation was represented by a very small number of cases [9,10]. During the 2013–2016 period, two new GII.17 variants emerged and caused large outbreaks, mainly in Asian countries. Although GII.17 did not achieve the same level of spread as the GII.4 variant, it has been regarded as a strain with significant potential for further dissemination [11]. In Argentina, the clinical surveillance strategy for acute diarrhea cases involves the weekly reporting of the number of affected individuals admitted to healthcare facilities. Additionally, gastroenteritis outbreaks are reported immediately using specific data collection questionnaires. In both cases, these situations represent mandatory reporting events.

In 2024, according to the data from the monitoring of diarrheal disease notifications, an increase in the trend of reported outbreaks was observed beginning in epidemiological week 26 (end of June) in three provinces. The aim of this study is to describe the epidemiological and genetic characteristics of three norovirus outbreaks reported in Argentina that were linked to GII.17 noroviruses.

## 2. Materials and Methods

In June and July of 2024, three outbreaks (A, B, and C) of gastroenteritis were reported in three different Argentine provinces. An epidemiological investigation was conducted of the three outbreaks using data collection forms. These questionnaires gathered information on the number of cases, the location of occurrence, symptoms, and the number of hospitalized individuals, among other relevant variables. Fecal or vomit samples from each of the three outbreaks were collected. After testing negative for bacterial enteropathogens, the samples were screened for rotavirus, norovirus, enteric adenovirus, sapovirus, and astrovirus using previously described molecular methods [12,13,14,15,16]. Norovirus-positive samples were genotyped based on the nucleotide sequence of the partial RdRp and capsid regions [17]. Near-full-genome amplification was performed on a representative sample from each outbreak, following the protocol described by Parra et al. [18]. Subsequently, whole-genome sequencing libraries were prepared from PCR amplicons by using the Illumina CovidSeq kit (San Diego, CA, USA), following the manufacturer’s instructions. Paired-end Illumina sequencing was performed on a NovaSeq 6000 platform (San Diego, CA, USA) (2 × 150 bp). The resulting FASTQ files underwent quality control analysis with FastQC [19], Trim Galore (http://www.bioinformatics.babraham.ac.uk/projects/trim_galore/, accessed 21 August 2024), and Kraken2 [20] using the Standard-8 database. After trimming, reads were mapped against all GII genomes present in the Human Calicivirus Typing Tool database (https://calicivirustypingtool.cdc.gov, accessed 21 August 2024) [21] using a multi-reference read mapper (in-house script, available at https://github.com/FacundoCuba/murema, accessed 21 August 2024), generating a consensus sequence with the closest reference for each sample.

Subsequently, phylogenetic analyses were conducted on the genes encoding the viral polymerase and capsid, along with a nucleotide and amino acid identity analysis between the GII.17[P17] strains detected in this study and other globally relevant strains available in GenBank. These strains correspond to different time periods in which different GII.17 variants were detected: (i) early 2000s (SaitamaT87, 2002); (ii) mid-2000s (Katrina, 2005); (iii) mid-2010s (Kawasaki323, 2014); (iv) mid-2010s that showed amino acid insertions (Kawasaki308, 2015) and; (v) sequences recently reported, associated with gastroenteritis outbreaks and sporadic cases detected between 2021 and 2024 in several countries. Alignments were performed using BioEdit v7.0.1. The base substitution model was estimated using the Model Finder module from the IQ-TREE web server according to the Akaike Information Criterion. Maximum Likelihood phylogenetic trees were constructed using the IQ-TREE web server, with TIM2 and GTR as substitution models for the capsid and polymerase sequences, respectively, and gamma-distributed rate variation among sites [22], and were visualized in iTOLv7 [23]. Ultrafast Bootstrap approximation (UFBoot; 10,000 replicates) was employed for phylogenetic branch support [24]. Identity analyses were conducted using MEGA X software (version 11.0.13).

The three near-full-genome sequences from this study were submitted to GenBank and assigned the following accession numbers: PQ482378–PQ482380.

## 3. Results

Epidemiological questionnaires revealed that the three outbreaks occurred in different provinces. Outbreak A was identified following 242 cases of diarrhea in the population, exceeding the expected number according to the endemic channel, with most cases occurring in adults. The main symptoms included vomiting, diarrhea, and abdominal pain. Two individuals required hospitalization for intravenous rehydration, but no fatalities were reported. Outbreak B occurred during a private event, where 23 cases of gastroenteritis were registered, predominantly affecting adolescents. Symptoms included vomiting, diarrhea, and abdominal pain. Two individuals required hospitalization, but no deaths were reported. Outbreak C involved a group of adolescents staying at a hotel during a school trip. Fifty cases were documented, with vomiting and diarrhea being the predominant symptoms. No hospitalizations or fatalities were reported in this case. For outbreaks A, B, and C, nine, five, and four clinical samples were received for analysis, respectively. The samples were mostly stool specimens, except in outbreak C, where only vomit samples were submitted. Norovirus GII was detected in seven out of nine samples in outbreak A, in four out of five samples in outbreak B, and in three out of four samples in outbreak C. All samples tested negative for the remaining viral agents. Norovirus-positive samples were genotyped based on the capsid region and RdRp, indicating that all detected norovirus strains belong to genotype GII.17[P17]. Additional clinical and epidemiological data from these outbreaks are summarized in Table 1.

**Figure 1:**
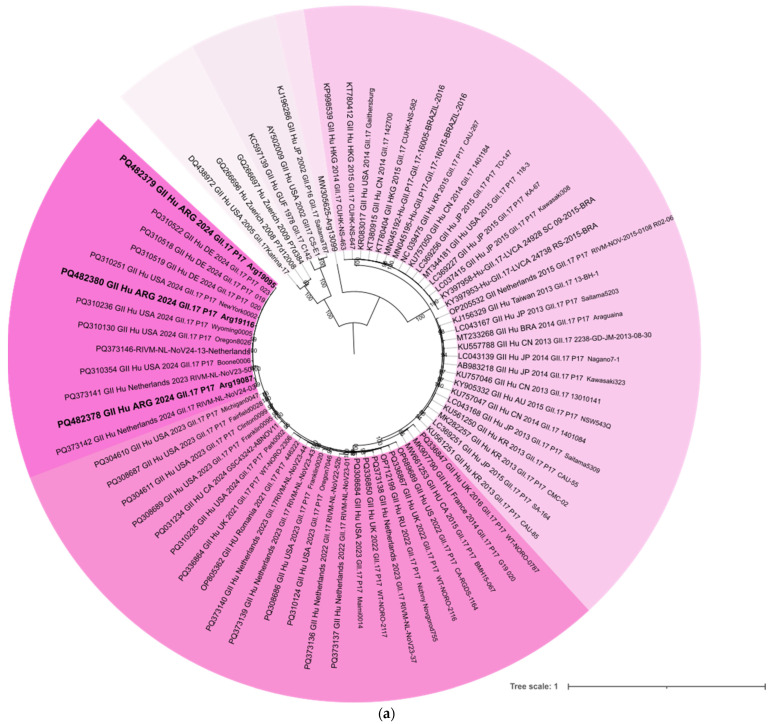

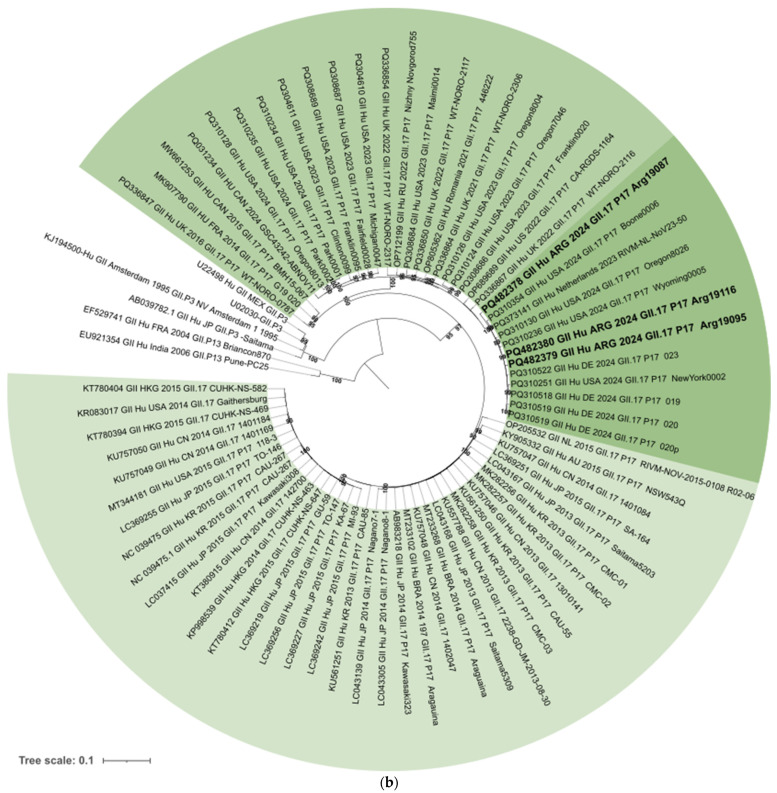
Phylogenetic analysis of GII.17[P17] strains detected in Argentina, 2024. Maximum Likelihood phylogenetic trees were constructed using the IQ-TREE web server for (**a**) VP1 (major capsid) and (**b**) RdRp (polymerase). For VP1 (1530 bp), the TIM2 substitution model with gamma-distributed rate variation among sites was used. Argentine strains are highlighted in bold within a cluster shaded in dark magenta; the grouping sequences reported are from between 2021 and 2024. Other lineages reported prior to 2020 (early 2000s, mid-2000s, and mid-2010s) are shaded in various shades of magenta. (**b**) For RdRp analysis (1623 bp), the GTR substitution model with gamma-distributed rate variation among sites was applied. Argentine strains, highlighted in bold, that are more closely related to contemporary P17 strains are shown in dark green, whereas other P17 strains that circulated in the mid-2010s are shaded in light green. Ten thousand Ultrafast Bootstrap replicates were employed for phylogenetic branch support. The GenBank accession numbers of all strains are shown with each sequence.

The phylogenetic analysis of the complete VP1 sequence revealed that Argentine strains were most closely related to strains circulating in Germany in 2024, as well as some from the United States and the Netherlands that circulated between 2023 and 2024 (Figure 1a). The comparative identity analysis of the representative strains selected from each of the three outbreaks revealed nucleotide and amino acid identity levels of 98.7% and 99.7%, respectively. Furthermore, a comparison of these Argentine strains with recently circulating strains in the USA and Europe showed nucleotide identity levels ranging from 96.4% to 99.2% and amino acid identity levels from 98.4% to 99.8% (Table 2). Consistent with the phylogenetic tree topology, the highest levels of identity were observed between the local Argentine strains and those identified in Germany. Additionally, among all strains detected from the 2020s onward, the number of amino acid substitutions varied from none to eight, with the highest proportion occurring in the P2 region. No deletions or insertions were observed. When the Argentine strains were compared to those detected in the mid-2010s specifically, they showed 93.2% nucleotide and 96.2% amino acid identity levels with the Kawasaki323 strain, and 92.2% nucleotide and 94.4% amino acid identity levels with the Kawasaki308 strain. The number of amino acid substitutions between the Argentine strains and mid-2010 strains ranged from 15 to 30, with the majority located in the P2 region. Notably, Kawasaki308 exhibited four insertions within the P2 region (Supplementary Materials).

Furthermore, the analysis of the complete RdRp genome sequence indicated that Argentine strains were most closely related to all contemporary P17 strains, clustering separately from P17 strains that circulated during the mid-2010s (Figure 1b). Even within acceptable confidence levels, the clustering pattern of the polymerase in the Argentine strains closely resembled that observed for the capsid.

## 4. Discussion

Through specific vaccination programs, the substantial decline in rotavirus-associated diarrhea cases has brought noroviruses into the spotlight as a key focus of attention over the past two decades. Additionally, increased access to molecular techniques in developing countries has led to improved etiological detection, providing a better understanding of acute diarrhea epidemiology. The detection of norovirus in this study coincided with a nationwide rise in acute diarrhea cases, observed both in sporadic instances and in the number of reported outbreaks. Although causality between the virus and the increase in cases cannot be established, it is recommended to reinforce active surveillance and broaden the search for various pathogens whenever a rise in cases or outbreaks is observed. It is important to note that in one of the outbreaks (outbreak C), the samples received consisted solely of vomit, as vomiting was the primary symptom among affected individuals. This situation highlights the need to reconsider case classification criteria, given that gastroenteritis presentations often manifest as vomiting without accompanying diarrhea. Such presentations can significantly influence surveillance data, introducing bias and potentially leading to the under-reporting of cases. Revisiting and refining classification criteria may enhance the accuracy of case recording and improve the reliability of surveillance systems. Increased diagnosis and a better understanding of norovirus’s role in gastroenteritis have generated growing global interest in the virus, its characteristics, and its genetic diversity. For example, while four genotypes (GII.2, GII.3, GII.4, and GII.6) were historically considered the most prevalent in humans, the GII.17[P17] genotype emerged as a prominent strain in the mid-2010s [11,25]. In contrast, Argentina-specific studies have reported that the GII.17 genotype has been detected only sporadically [26,27]. A sequence analysis of the amino acid composition of the major capsid protein revealed significant differences, particularly in the P2 hypervariable region, between GII.17 viruses circulating and predominating in the mid-2010s and those described in this study and recently circulating in Germany, the USA, and the Netherlands during the 2022–2024 period [28]. This finding suggests that the identified genotype might represent a new lineage. Although the cutoff for distinguishing norovirus genotypes is defined as a 15% difference in the amino acid sequence of the complete VP1 gene, there is currently no standardized criterion for defining novel variants [6]. For example, the classification of GII.4 variants has shifted to relying on phylogenetic clustering patterns rather than strict sequence divergence. In this context, new GII.4 variants are typically recognized as demonstrating widespread circulation across at least two geographically diverse regions and as demonstrating associated epidemiological significance [5]. Similarly, the phylogenetic clustering pattern observed in this study reinforces the distinctiveness of the identified GII.17 strains, supporting the proposal of them as a novel lineage and highlighting their potential epidemiological relevance. Additionally, the strains detected in Argentina exhibit high genetic similarity to strains reported in the Northern Hemisphere beginning in 2021. To the best of our knowledge, this represents the first report of this potential novel variant in the Southern Hemisphere, as these strains differ from the GII.17 strains previously reported in the region [26,29,30,31]. Among recent GII.17 genotypes, the Argentine strains from this study were found to resemble a subgroup of those from the United States, despite the latter displaying significant heterogeneity in amino acid substitutions. This could be attributed to the virus’s own evolutionary dynamics [18]. Therefore, as more sequence data for this genotype become available, phylodynamic studies would be valuable to investigate whether these differences are linked to distinct patterns of dissemination or are the result of rapid viral evolution. As highlighted over a decade ago, the evolutionary dynamics and circulation patterns of the GII.17 genotype appear to mirror those of GII.4, characterized by the periodic emergence of new variants [11]. Furthermore, the analysis of the phylogenetic tree revealed that the P.17 polymerase genotype identified in this study clustered differently from sequences reported during the 2013–2015 period. Thus, future research should explore whether interactions with the ORF1 polyprotein influence the adaptation of the VP1 protein, as suggested by these genotype combinations. It remains to be determined if the observed amino acid variations, compared to previously circulating lineages, have antigenic differences that could potentially enable this strain to evade immune responses in populations with prior exposure to this genotype. Effective public health surveillance and diagnostic strategies are essential, as timely etiological diagnosis enables the monitoring of disease burden trends and helps to interrupt transmission chains. For instance, during the SARS-CoV-2 pandemic, the rapid implementation of real-time diagnostic capacity facilitated the identification of hotspots, informed public health interventions, and supported efforts to break transmission chains [32,33]. Enhanced real-time detection capabilities improved the ability to rapidly identify emerging pathogens, monitor their evolution, and implement targeted control measures to mitigate outbreaks effectively. Gastroenteritis surveillance presents particular challenges due to the self-limiting nature of most cases, which often do not require hospitalization and can obscure the true incidence, as well as the variety of agents involved. Thus, alternative tools, such as passive epidemiological surveillance and virological detection in environmental samples, could serve as useful predictive indicators. Nonetheless, there is an ongoing challenge and responsibility to promote training and improve access to timely etiological diagnosis and genomic surveillance, especially among key community transmission groups such as food handlers or children. Viral infections related to acute gastroenteritis remain a major public health concern across all age groups, with incidence unrelated to the socioeconomic level of countries or regions. Therefore, it is crucial to continue studies that characterize the global disease burden to inform the development of optimal prevention and control strategies.

## Figures and Tables

**Table 1:** Epidemiological and clinical characteristics and outcomes of outbreaks A, B, and C.

Outbreak	Location	Date (Onset)	Date (End)	Setting	Symptoms *	No. of Exposed Individuals	No. of Cases	Age Average	Age Range	No Hospitalizations	No Deaths
A	Villa La Angostura, Neuquén	25 June 2024	5 July 2024	Local community	D + V + AP	15,552	242	Adults and children	6 months–64 years	2	0
B	Carlos Casares, Buenos Aires	1 July 2024	4 July 2024	Social event	D + V + AP + F	106	23	Children and adolescents	2 years–17 years	2	0
C	San Carlos de Bariloche, Río Negro	15 July 2024	2 August 2024	Hotel	V + D	1500	179	Adolescents	17 years–18 years	0	0

* The reported symptoms included the following: D (diarrhea), V (vomiting), AP (abdominal pain), and F (fever).

**Table 2:** Comparison of genetic characteristics of GII.17[P17] norovirus strains detected in Argentina (2024) and globally reported strains.

Variant	Country or Strain Name	Year/Period of Detection	Identity nt ^b^ (%)	Identity aa ^c^ (%)	Number of aa Substitutions Observed in VP1	Number of aa Substitutions Observed in P2 Region (#, %)	Insertions/Deletions
2020s onward	Canada	2024	96.6	98.6	7	4 (57.1%)	No insertions or deletions
	**Germany** ^a^	**2024**	**99.2**	**99.8**	**0**	**n/a** ^d^	No insertions or deletions
	Netherlands ^a^	2023–2024	97.3	99.2	4	3 (75.0%)	No insertions or deletions
	Romania	2021	96.4	98.7	6	4 (66.7%)	No insertions or deletions
	Russia	2022	96.4	98.4	8	5 (62.5%)	No insertions or deletions
	United Kingdom ^a^	2021–2022	95.9	98.7	6	5 (83.3%)	No insertions or deletions
	USA ^a^	2022–2024	97.3	99.3	2	1 (50.0%)	No insertions or deletions
Mid-2010s	Kawasaki323	2014	93.2	96.2	15	11 (73.3%)	No insertions or deletions
	Kawasaki308	2015	92.2	94.4	30	25 (83.3%)	4 insertions ^e^
Mid-2000s	Katrina	2005	77.1	87.2	68	43 (63.2%)	4 insertions ^f^
Early 2000s	SaitamaT87	2002	76.1	89.1	56	35 (62.5%)	4 insertions and 2 deletions ^g^

The strains (or group of strains) that showed the highest identity at the nucleotide and amino acid levels are in bold. ^a^ As these strains showed intragroup variability, the most represented amino acids were considered for variable sites. ^b^ nt: nucleotide. ^c^ aa: amino acid. ^d^ n/a: not applicable. ^e^ Insertions = +2 between 371 and 372 and +2 between 388 and 389. ^f^ Insertions = +3 between 293 and 294 and +1 between 378 and 379. ^g^ Insertions = +3 between 293 and 294 and +1 between 378 and 379; deletions = positions 340–341.

## Data Availability

The GenBank accession numbers for the three sequences obtained for this study are OM339145-OM339149.

## Supplementary Materials

The following supporting information can be downloaded at https://www.mdpi.com/article/10.3390/v17020223/s1, Table S1 Amino acid sequence positions of the VP1 protein (capsid) in GII.17[P17] norovirus strains detected in Argentina (2024) and globally reported strains.

## Abbreviations

The following abbreviations are used in this manuscript:

AGE	sporadic acute gastroenteritis
ORF	open reading frames
RdRp	RNA-dependent RNA polymerase
