# Supplementary material for: Multi-Province Outbreak of Acute Gastroenteritis Linked to Potential Novel Lineage of GII.17 Norovirus in Argentina in 2024
Source: Viruses. 2025 Feb 5;17(2):223. doi: 10.3390/v17020223 (PMC11860177; doi:10.3390/v17020223)
Supplement: Supplementary file 1 [file viruses-17-00223-s001.zip › Supplementary file S1 legends.pdf]

Supplementary file S1. Amino acid sequence positions of the VP1 protein (capsid) in GII.17[P17] norovirus strains detected in Argentina (2024) and globally reported strains. Supplementary file provides a detailed comparison of the amino acid sequence positions of the VP1 protein (capsid) in GII.17[P17] norovirus strains detected in Argentina in 2024 and other GII.17 strains reported worldwide across different time periods. It includes information on amino acid variations and their specific positions.
